# Supplementary material for: Development of a Molecular Aptamer Beacon Applied to Magnetic-Assisted RNA Extraction for Detection of Dengue and Zika Viruses Using Clinical Samples
Source: Int J Mol Sci. 2022 Nov 10;23(22):13866. doi: 10.3390/ijms232213866 (PMC9693377; doi:10.3390/ijms232213866)
Supplement: Supplementary file 1 [file ijms-23-13866-s001.zip › ijms-1956186-supplementary.pdf]

Supplementary Information

Methods & Results

Evaluation of the bifunctionality of post-Selex aptamers: A pool of biotinylated ssDNA from the 5'UTR of the DENV of the four viral serotypes and 5'UTR of ZIKV, obtained by asymmetric PCR (bio-ssDNA), were separately denatured at 95°C for 10 minutes and then incubated for 15 minutes with 1μM of aptamers. After the incubation period, paramagnetic particles coupled with streptavidin were added, which allowed the immobilization of biotinylated molecules. To remove non-binding molecules, a magnetic platform was used, and the binding molecules were eluted in 10 μl of ultrapure water. The aptamers eluted in the capture were amplified by Real-Time PCR. The reaction was performed under the following conditions: 2 μL of the captured sample, 1X of the qPCR Mix buffer, 2.5 μM of each primer (forward and reverse) and completed with ultrapure water for a final volume of 10 μL. The melting curve for each one of fourteen aptamers was analyzed after amplification. Duplicates were made for each captured aptamer; the data were evaluated according to the Cts (Cycle Threshold) and the melting curves (TM) (Figure S1).

Analysis of the influence of ions present in the buffer: The effect of ions was studied separately for each of the cations present in the binding buffer, the buffer used for the selection of the aptamers (20 mM Tris-HCl, pH 7.4, 100 mM NaCl, 4 mM $MgCl_2$, 5 mM KCI, 30 mM $CaCl_2$). Different concentration ranges were used for each of the analyzed species: K^+ (0- 10 mM), Na^+ (0 - 500 mM), Ca^{+2} (0 - 6 mM) and Mg^{+2} (0 - 5 mM). The assays were carried out at 25 ºC and with 50 nM of aptamer APTAZC10-MB in 20 mM Tris pH 7.4. The results obtained were analyzed using the ratio of the fluorescence emission intensity of the TAMRA to the FAM intensity, F_{580} / F_{520} (Figure S2).

Analysis of the temperature effect: 50 nM of the aptamer APTAZC10-MB were submitted to temperatures varying from 25 ºC to 85 ºC, in 20 mM Tris pH 7.4 with and without the presence of the target, 100 nM 5 'UTR-ZIKV (Figure S3). The relative efficiency of fluorescence resonance energy transfer upon temperature change was determined by the following equation:

$$E = \frac{I_a}{I_a + I_d}$$

Where E is the relative efficiency of FRET, I_a and I_d are the total fluorescence intensities of the acceptor (TAMRA) and the donor (FAM), respectively, both after FAM excitation.

Inoculation of the serum samples in C6/36 cells: 5x 10^5 C6/36 cells (*Aedes albopictus*) were seeded in a 24-well plate and grown in L-15 medium containing penicillin and streptomycin (10,000 U / mL and 10,000 μg / mL), supplemented with 1% non-essential amino acids, 10% tryptose phosphate broth and 5% fetal bovine serum (SFB), and kept at 28°C. A 100 μL volume of the serum sample from a patient positive for ZIKV was incubated for 1 hour at 28°C for adsorption. The cells were subsequently kept in L-15 medium supplemented with 2% SFB (Figure S4). After 7 days, the cytopathic effects (CPE) were investigated. The formation of the syncytia in the cells occurred with inoculation of the serum but was not verified in the C6/36 without inoculation, which was used as a negative control. The RNA was extracted by TRI Reagent® (Sigma-Aldrich) according to the manufacturer's instructions. Subsequently, RT-PCR reactions were performed, and the material obtained was analyzed by electrophoresis (Figure S5), presenting a diffuse band profile expected for ZIKV in agarose gel.

FIGURES

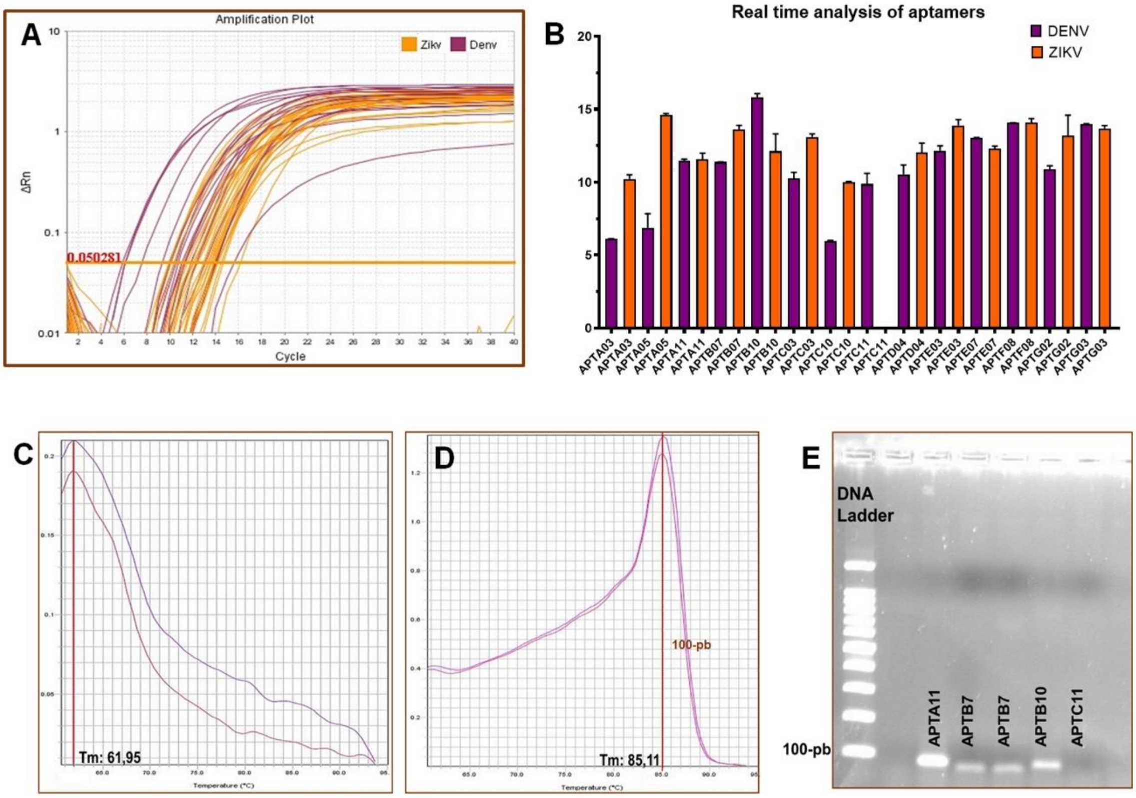

Figure S1. Real-time PCR assay with aptamers captured by the targets. (A) The curve of amplification: in orange-aptamers captured by 5'UTR-ZIKV; in purple-aptamers captured by 5'UTR-DENV. (B) Cycle threshold (Cts) analysis for fourteen aptamers captured by each bio-ssDNA viral sequence. Different Cts were observed for DENV compared with ZIKV. Interaction of ZIKV and APTC11 was not observed, thus indicating this aptamer to be specific to the 5'UTR-DENV. The lowest Ct for both flaviviruses was observed for the APTAZC10 aptamer, corroborating the NGS data analysis presenting a higher number of hits of possible interaction with the target, and this ligand was chosen for subsequent characterization tests. (C) Melting curve of the negative reaction control. (D) Melting curve of the aptamers. Figures C and D indicated no primer dimers. The melting temperature of aptamers was 85.11°C. (E) Agarose gel electrophoresis of the preliminary post-Selex aptamer capture assay by bio-ssDNA 5'UTR-ZIKV. These data are corroborated by a preliminary capture test for APTC11, where aptamer amplification through conventional PCR was not observed.

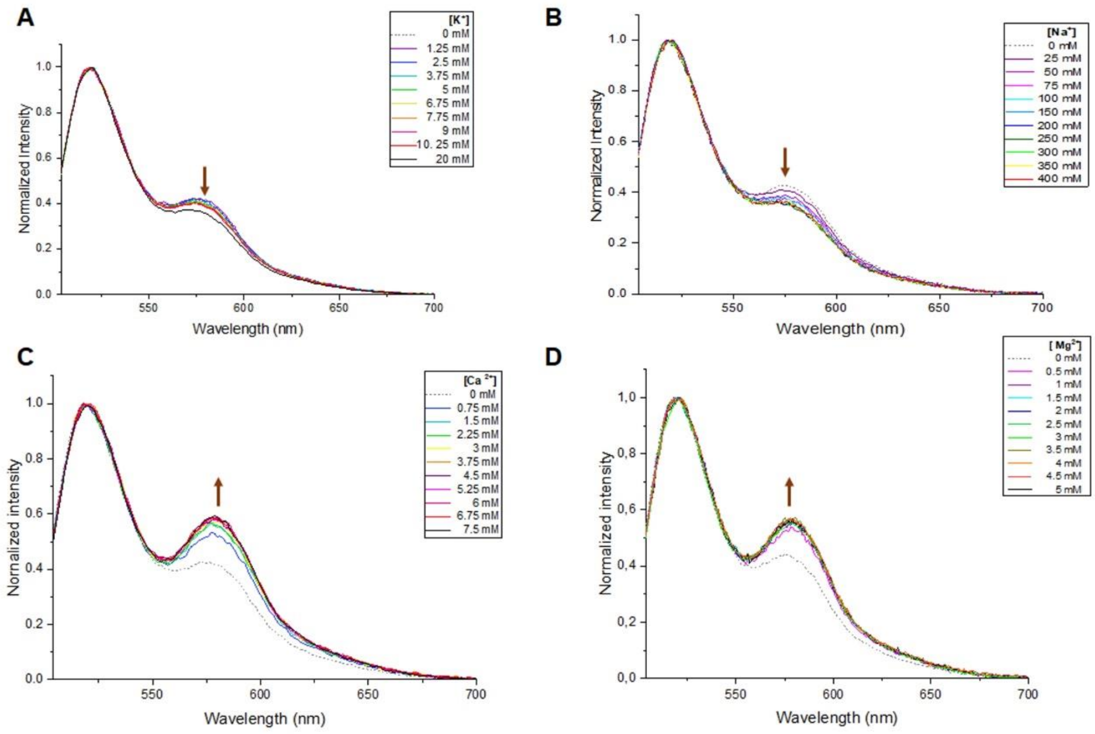

Figure S2: Effect of ions on the structural conformation of the aptamer APTAZC10-MB. The ions analyzed in the binding buffer – namely K^+ (A), Na^+ (B), Ca^{2+} (C) and Mg^{2+} (D) – were divalent ions presenting greater effect on the aptamer molecules.

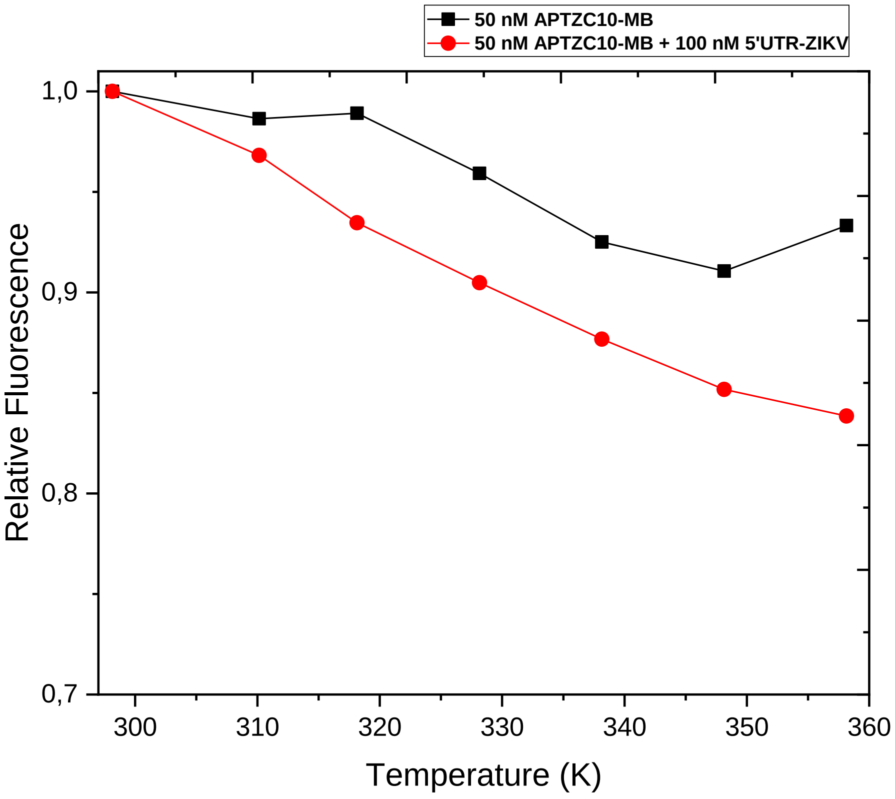

Figure S3: Study of the influence of temperature on the efficiency of FRET with and without the presence of the 5′UTR-ZIKV. The temperature increase causes the rupture of hydrogen bonds, which distances the fluorophores, FAM and TAMRA, thus increasing the intensity of the donor and decreasing FRET efficiency. The aptamer together with the target presented a more expressive change in the molecule as an effect of bimolecular interactions.

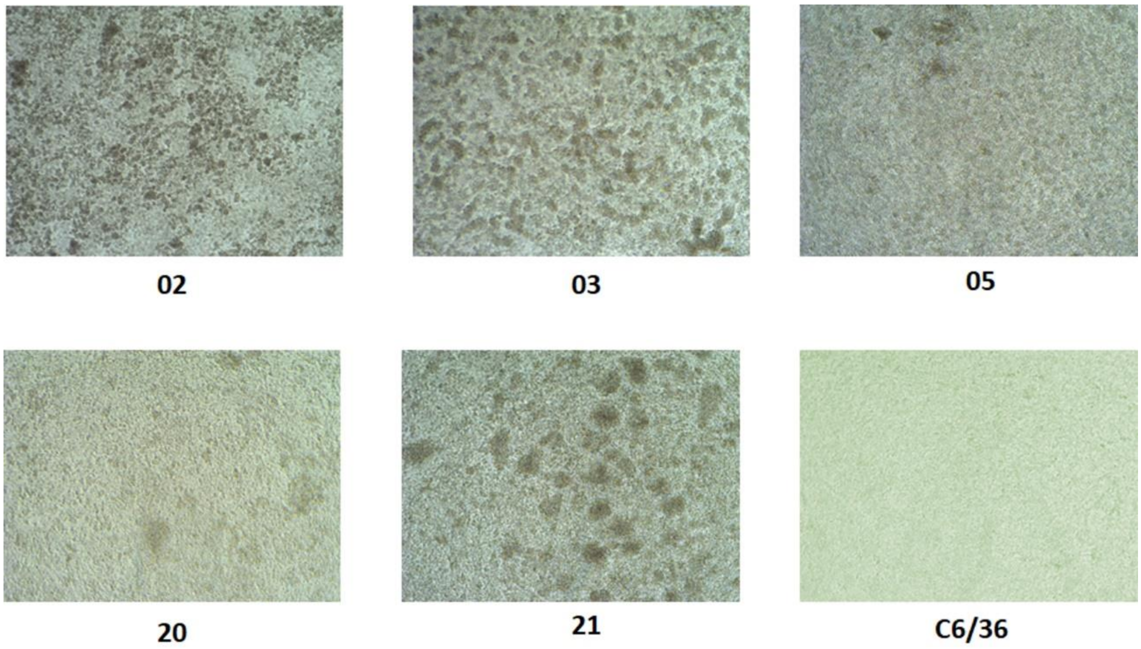

Figure S4: C6/36 cells 7 days after inoculation of the five patient samples (02, 03, 05, 20, and 21) suspected to be infected by ZIKV. It is possible to observe the formation of the syncytia in the cells with inoculation of the patient samples while it was not verified in the C6/36 cells without inoculation, which were used as a negative control.

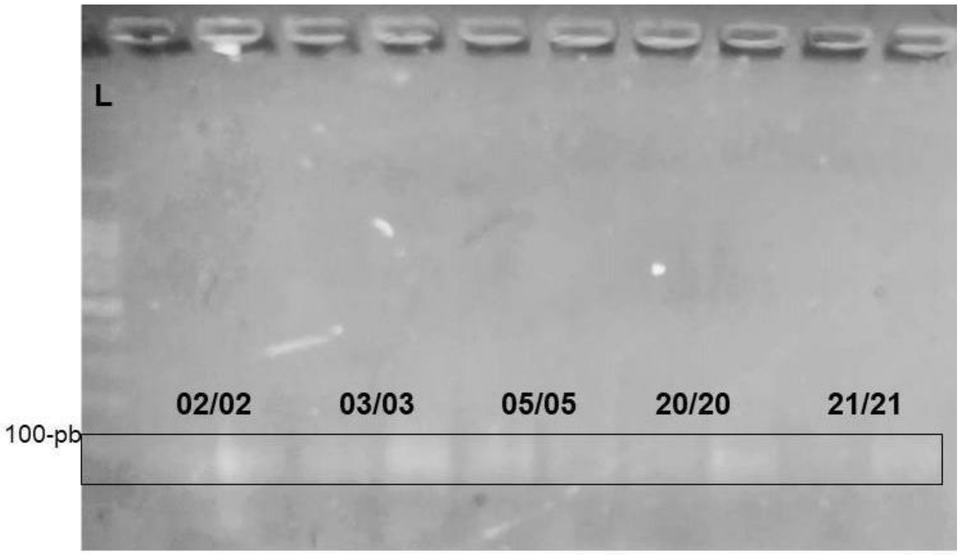

Figure S5: Electrophoresis of the products obtained by RT-PCR of RNA extracted from infected cells with serum patients L- Ladder; 02, 03, 05, 20, and 21 suspected cases of ZIKV infection. Due to the weakly obtained bands, sequencing of the products should have been performed to confirm ZIKV.

TABLES

Table S1. Diagnostic parameters of the APTA-RT-PCR for DENV detection in comparison with serological data.

**Laboratory tests**	**Diagnosis Parameters APTA-RT-PCR % (IC 95 %)**			
	**Sensibility**	**Specificity**	**PPV**	**NPV**
**Immunodiagnostics (IgM+IgG+NS1)**	52 (38 to 67)	52 (39 to 64)	44 (32 to 58)	59 (46 to 71)
**IgM**	35 (21 to 53)	56 (37 to 73)	50 (30 to 69)	41 (26 to 58)
**IgG**	38 (23 to 56)	63 (44 to 78)	52 (32 to 72)	49 (33 to 64)
**NS1**	63 (39 to 82)	59 (45 to 72)	33 (19 to 51)	83 (67 to 92)
**Platelets**	60 (31 to 83)	57 (43 to 70)	22 (11 to 41)	88 (72 to 95)

PPV = positive predictive value; NPV = negative predictive value.

Table S2. Correlation analysis between immunodiagnostics and molecular DENV detection.

Laboratory tests	Apta-RT-PCR	
	Positive	Negative
**Platelets**		
< 150,000	10.17%	36.60%
≥ 150,000	6.78%	47.46%<br>p= 0.48
**White Corpuscles**		
<4000	11.86%	33.90%
≥4000	20.34%	33.90% p=0.41
**IgM**		
Positive	19.64%	35.71%
Negative	19.64%	25%<br>p= 0.58
**IgG**		
Positive	21.42%	30.36%
Negative	17.86%	30.36% p=0.78
**NS1**		
Positive	15.38%	9.23%
Negative	30.77%	44.62% p=0.15
**Immunodiagnostics**		
IgM+IgG+	20.93%	32.56%
IgM-IgG-	18.60%	27.91%<br>p= 1.00
**Immunodiagnostics**		
IgM+IgG+	30%	46.67%
IgM+IgG-	6.67%	16.67% p=0.69
**Immunodiagnostics**		
IgM+IgG+	31.03%	48.28%
IgM-IgG+	10.34%	10.34% p=0.67
**Immunodiagnostics**		
IgM+IgG-	7.41%	18.52%
IgM-IgG-	29.63%	44.44% p=0.68
**Immunodiagnostics**		
IgM-IgG+	11.54%	11.54%
IgM-IgG-	30.77%	46.15% p=1.00

p value: Fisher’s exact test.
